# Informed consent for MRI and fMRI research: Analysis of a sample of Canadian consent documents

**DOI:** 10.1186/1472-6939-12-1

**Published:** 2011-01-14

**Authors:** Nicole Palmour, William Affleck, Emily Bell, Constance Deslauriers, Bruce Pike, Julien Doyon, Eric Racine

**Affiliations:** 1 Neuroethics Research Unit, Institut de recherches cliniques de Montréal, (110 avenue des Pins Ouest), Montréal, (H2W 1R7), Canada; 2 Department of Preventive and Social Medicine (Bioethics Programs), University of Montreal, (2375 chemin de la Côte-Ste-Catherine), Montréal, (H3T 1A8), Canada; 3 Biomedical Ethics Unit, McGill University, (3647 Peel Street), Montréal, (H3A 1X1), Canada; 4 Department of Neurology and Neurosurgery, McGill University, (3801 University Street), Montréal, (H3A 2B4), Canada; 5 Department of Psychology and Functional Neuroimaging Unit, University of Montreal, (4545 chemin Queen-Mary), Montréal, (H3W 1W5), Canada; 6 Division of Experimental Medicine, McGill University, (1110 Pine Avenue West), Montréal, (H3A 1A3), Canada

## Abstract

**Background:**

Research ethics and the measures deployed to ensure ethical oversight of research (e.g., informed consent forms, ethics review) are vested with extremely important ethical and practical goals. Accordingly, these measures need to function effectively in real-world research and to follow high level standards.

**Methods:**

We examined approved consent forms for Magnetic Resonance Imaging (MRI) and functional Magnetic Resonance Imaging (fMRI) studies approved by Canadian research ethics boards (REBs).

**Results:**

We found evidence of variability in consent forms in matters of physical and psychological risk reporting. Approaches used to tackle the emerging issue of incidental findings exposed extensive variability between and within research sites.

**Conclusion:**

The causes of variability in approved consent forms and studies need to be better understood. However, mounting evidence of administrative and practical hurdles within current ethics governance systems combined with potential sub-optimal provision of information to and protection of research subjects support other calls for more scrutiny of research ethics practices and applicable revisions.

## Background

Research ethics and the measures deployed to ensure ethical oversight of research (e.g., informed consent forms, ethics review processes) are vested with extremely important goals.[1] First and foremost, research ethics aims to protect human subjects and inform them of their rights and of the risks related to their participation in a research project. This has been a tenet of modern research ethics for decades and is consistent with the inescapability of individual rights and respect for autonomy in contemporary research. Second, as a result, research ethics oversight makes it possible for research to be carried on in contemporary social and political environments where the failure to comply with basic norms of ethics and individual rights can lead to severe backlash on researchers, academic institutions, research communities, and funding bodies. Hindered research also translates into opportunity costs because the potential development of novel treatments and the beneficial outcomes of research can be lost when unfortunate research ethics events occur.[2,3] This is in addition to precedent skids and their consequences that we still live with. Therefore, all stakeholders in research have a vested interest in attending diligently and seriously to research ethics and human subject welfare in order to ensure the continuity of the research enterprise and to harness support for the deliverables of research.

To fulfill these important goals, the measures put in place to deal with research ethics, issues such as informed consent forms and ethics review need to work effectively in real-world research and to follow high level standards.[4] Fulfilling the goals of informing and protecting subjects, as well as ensuring the continuity of research, rely in large part on the ability of these measures to respond to current and future research ethics challenges. Accordingly, many have called for further evidence-based research ethics practices in the light of apparent knowledge gaps and disquieting practical problems of ethics review.[5,6] In this paper, we report an examination of consent forms approved by Canadian Research Ethics Boards (REBs). We were interested in comparing consent forms and understanding how they met (or not) basic requirements of the established Canadian Tri-Council Policy Statement (TCPS) as well as how they dealt with emerging issues in neuroscience research. As part of a broader study, we focused on neuroimaging research because Magnetic Resonance Imaging (MRI) and functional Magnetic Resonance Imaging (fMRI) research techniques often fit minimal risk criteria[7] but have, nevertheless, sparked debates related to the discovery of incidental findings[8,9] in research as well as to the capabilities of neuroprediction of personality and behaviour.[10,11]

## Methods

Neuroimaging researchers participating to a broader study on the challenges of ethics review in the Canadian context were invited to submit their two most recently approved informed consent forms in English or in French. Consent forms for other techniques than MRI and fMRI were excluded to focus the sample and analysis (n = 23). Data was collected from 2008 to 2009.

### Study Design

All forms were coded or parsed based on a standard thematic qualitative content analysis approach.[12] A coding guide was elaborated to support the analytic process. This guide identified and defined the primary, secondary and tertiary level codes and included examples for each code to guide coding. This guide was drafted on the basis of the analysis of a diversified sample of consent forms where free nodes were generated in an *open-coding *phase. The coding guide was developed to capture the content required for consent documents as described in the first edition of the TCPS.[13] The TCPS constitutes the official research ethics policy of the three major Canadian funding bodies: the Canadian Institutes of Health Research (CIHR), the Natural Science and Engineering Research Council of Canada (NSERC), and the Social Sciences and Humanities Research Council of Canada (SSHRC). All researchers and institutions receiving funds from these bodies must comply with the TCPS and, de facto, the TCPS has become the "national" Canadian policy of reference even though it has limited jurisdiction. Piloting and test coding were done on a small sub-sample of forms to ensure validity and consistency. Ethical issues not explicitly addressed in the TCPS but identified in the pilot sample as featured in the consent forms were added to the coding guide. Specifications on how consent forms dealt with TCPS requirements were also captured. Preliminary versions of the coding guide (drafted by WA) and of the pilot coding were reviewed and discussed (by WA, NP and ER) before final coding was conducted.

### Data Analysis

All informed consent documents were then systematically coded (by NP and WA), i.e., the *axial coding *phase, and a final review of the coding was conducted by some of the investigators of the present study (by EB, CD, and ER). The final coding guide included the following topics: (1) general conditions of informed consent required by the current TCPS [13] (e.g., statement of the research purpose, identity of the researcher; Section D, Article 2.4, pages 2.5-2.6 of TCPS); (2) additional items of informed consent that may be required by REBs depending on the nature of the study (e.g., statement that new information about the study will delivered to the subject in a timely manner, measures to protection confidentiality) (Table 1, section D, page 2.7 of TCPS) and (3) a salient issue in the ethics of neuroimaging research: incidental findings. All coding was supported by the use of the QSR NVivo 7 and 8 qualitative analysis software packages (Doncaster, Australia). REB approvals were obtained prior to the beginning of this study for each targeted site. Results are reported using descriptive statistics to quantify the presence of coded items in consent forms. Qualitative data is used to report salient aspects and strategies employed to deal with ethical issues.

**Table 1:** TCPS general conditions of informed consent found in approved consent forms

General conditions of informed consent according to the TCPS	n	%
A. Individual invited to research project*	**32**	**74%**
B. Statement of research purpose, identity of designated researcher, expected duration of study, description of research procedures*	**43**	**100%**
Statement of research purpose	43	100%
Expected duration of study	43	100%
Description of research procedures	43	100%
Screening procedures	39	91%
Identity of designated researcher	41	90%
C. Description of reasonably foreseeable harms and benefits, consequences of non-action*, **	**43**	**100%**
Potential risks	43	100%
Potential benefits	42	98%
D. Assurance of freedom not to participate, right to withdraw, identification of continuing and meaningful opportunities to continue to participate*	**43**	**100%**
Right to withdraw	43	100%
Freedom not to participate	39	91%
Continuing and meaningful opportunities to participate	11	26%
Consequences of non-action	36	84%
E. Conflict of interest and commercialization*	**9**	**21%**

*Categories bolded (A, B, C, D, E) indicate a general category of information required by the Canadian Tri-Council Policy Statement (TCPS).[13]

** "Consequences of non-action" designate the consequences of not being enrolled in the protocol. This aspect concerns more directly pharmacological trials or invasive trials.[13] See Table 3 for breakdown data on risks and benefits. "Benefits" also include claims about the non-existence of benefits.

## Results

We collected a total of 43 consent forms from 9 distinct Canadian sites featuring research designs based on anatomical MRI and functional MRI. Twenty-nine of the 41 (70.7%) researchers asked to submit their most recent consent forms participated (submitted consent forms).

### 1. General conditions required for informed consent

The TCPS requires that (by default) some general conditions be fulfilled in the written informed consent process such as (1) specifying that the individual is invited to a research project; (2) a statement of research purpose, the identity of designated researcher, the expected duration of study, and the description of research procedures; (3) description of reasonably foreseeable harms and benefits, and consequences of "non-action" (not participating to the protocol); (4) assurance of freedom not to participate, right to withdraw, identification of continuing and meaningful opportunities to continue to participate and (5) information on conflict of interest and commercialization (see bold items in Table 1).[13] In our sample, most of these items required by the TCPS were included in approved consent forms (Table 1). However, only three quarters of consent forms included a sentence specifying that the consent form was an invitation to research (e.g., "You are being asked to participate in a research study" or "You have been invited to take part in a research study").

All forms included descriptions of research procedures. Ninety one percent of informed consent forms contained information describing the imaging device, including 77% of forms describing physical aspects of the device and 35% of forms describing operational aspects. The TCPS requires that the expected duration of the study be presented and this was present in all consent forms. However, 98% stated the duration of each visit; 91% the duration of patient participation; and 28% mentioned the total duration of the study. Specifications about the right of subjects to withdraw from the study were encountered in all forms and the "consequences of non-action" as defined by the TCPS were found in most consent forms (e.g., "If you chose to stop being in the study, this will not affect your ongoing health care", "Refusal to participate will involve no penalty or loss of benefits to which you are otherwise entitled" or "You are free to discontinue participation at any time without prejudice to your subsequent care, and are free to seek care from a health care provider of your choice at any time"). Funding sources or sponsoring partners of the protocols were identified in 23% of consent forms. A general description of foreseeable risks was present in all consent forms and statements about benefits (e.g., absence of benefits or existence of possible benefits) in almost all (98%; Table 1).

Table 2 presents more detailed data on the conditions of information on potential harms (risks) and benefits found in the sample. The most widely listed benefit to participation in the informed consent documents related to contributing to a better understanding of brain function or brain dysfunction (72%). Some informed consent documents described that a participant could benefit personally from the detection of incidental findings (9%). Half the forms (53%) presented statements to dispel any misunderstanding that the research proposal would provide clinical benefits, i.e., a diagnostic or therapeutic misconception (e.g., "You should be aware that the X-Institute for Y is not a medical setting but a research organization. Also, you should know that the images that will be taken do not meet the criteria for clinical diagnostics" and "This is a research study so you will not personally benefit, clinically or financially, by participating in this study"). Physical risks, which were identified in 93% of informed consent forms included descriptions as follows: (1) the risks created by the presence of metal objects (implanted or not) (72%); (2) high noise levels (67%); (3) the risks of tissue burns (19%); (4) possible dizziness and or lightheadedness (19%); (5) physical discomfort (16%); and (6) stress and fatigue (16%). Risks related to sedation were described in 5% of the protocols but this procedure is not common to all neuroimaging research protocols. Certain categories of risks (e.g., pregnancy-related risks, psychological risks) were not addressed in all protocols. Statements about unidentifiable risks were included in only 33% of the consent forms.

**Table 2:** Description of foreseeable harms and benefits in approved consent forms*

	n	%
Potential benefits*	**42**	**98%**
Better understanding of brain function or dysfunction	31	72%
No personal benefit	27	63%
Future benefit to other patients	18	42%
Technical improvements to existing methods	8	19%
Clinical benefits	4	9%
Detection of incidental findings**	4	9%
Potential risks*	**43**	**100%**
Physical risks***	40	93%
Psychological risks****	24	56%
Unidentified risks	14	33%
No risks, minimal, risks or risk minimizing statement	39	91%
Risk-mitigating strategies	34	79%
Pregnancy-related risks	9	21%

* Categories in bold indicate a general category of information required by the Canadian Tri-Council Policy Statement (TCPS). Other rows report how consent forms dealt with the general conditions.

** Protocols described incidental findings as "neuro-abnormalities"

*** See text for breakdown data of physical risks

**** Psychological risks were almost entirely related to claustrophobia

Statements that there were "no risks" or statements which minimized risk were present in 91% of the analyzed consent forms despite infrequent but severe risks of injury associated with MRI and fMRI due to the magnetic field that can damage implanted metal devices or pull loose metal objects.[7] Many statements were moderate (i.e., "risk-minimizing statements") but some clearly stated that there were no risks involved:

"The fMRI experiment might be tiring but is not dangerous or invasive

(...) There are no risks involved in participating in any part of the study".

"There is no harm associated with the scanner and the process is painless".

"MRI scanners do not use radiation and are not known to be dangerous in any way".

Statements reporting strategies to mitigate risk were presented in 79% of the informed consent documents included. Some examples of these statements included the following:

• Actions researchers do to prevent risk (49%) (e.g., "MRI is noisy. In order to protect your hearing, we will provide you with ear plugs for your safety" and "To minimize anxiety associated with a feeling of claustrophobia experienced by some children during the extended study period inside the magnet, your child will be encouraged to enter the magnet for short periods to experience the sensation before providing their final consent to participate").

• Researcher responsibility in case of injury (28%) (e.g., "If you become ill or injured as a direct result of participating in this study, necessary medical treatment will be available at no additional cost to you").

### 2. Additional information that may be required by REBs

The TCPS indicates that a number of content items may be required by REBs depending on the nature of the study (Table 1 of TCPS document, page 2.7).[13] Many of these items were covered in a majority of consent forms (e.g., confidentiality in a broad sense, study compensation) but others appeared only in minority of approved consent forms (e.g., circumstances for termination of the study, explanation of the responsibilities of subjects) (Table 3). Study compensation took various forms: (1) reimbursement of expenses (47%); (2) compensation for time and participation (42%); (3) payment per hour or session (26%); (4) picture of subject's brain offered as compensation (21%); (5) no compensation offered (9%); (6) gifts offered (7%); (7) school credit (7%); (8) and food (2%).

**Table 3:** Additional information that may be required by REBs for informed consent according to the TCPS*

Information that may be required by REBs	n	%
Statement that new information about the study will be provided*	**13**	**30%**
**Identification of qualified representative to explain research**	**38**	**88%**
**Identification of appropriate resources outside research team for ethical issues**	**41**	**95%**
**Explanation of procedures of confidentiality and access to research data**	**43**	**100%**
Who has access to data	35	81%
Modalities for the storage of the data	34	79%
Confidentiality in the dissemination of research	31	74%
Disclaimer of non-guarantee of confidentiality	30	70%
Explicit statements about non-guarantee of confidentiality	15	35%
Implied non-guarantee of confidentiality	28	65%
Description of the methods to protect confidentiality of data	12	28%
**Description of circumstances for termination of subjects participation**	**18**	**42%**
No reason needed	15	35%
Subject's best interest	5	12%
Subject is unable or unwilling to comply with study protocol	3	7%
Description of study compensation***	**39**	**91%**
**Explanation of the ways in which results will be published**	**29**	**67%**
Ways research will be published	25	58%
Subject access to published results	11	26%

* Information that may be required based on Table 1 of the TCPS (Section D, 2.6)

Categories highlighted in bold indicate a general category of information of the Canadian Tri-Council Policy Statement (TCPS). Other rows report how consent forms dealt with the general conditions. We did not include items 5, 8 and 9 of Table 1 of the TCPS since they concern randomization and biomedical procedures and trials.

**New information about the study conveyed to subject if likely to change subject's decision to participate.

***See breakdown data in the text.

### 3. Management of incidental findings

In our sample, we encountered 13 distinct methods or strategies used by REBs to handle incidental findings (see Table 4). Strategies between sites varied in terms of their specific attributes, for example, on whether or not the subject was given the choice to be informed of possible incidental findings, and how or what medical specialists were, or were not, brought in to examine research scans upon discovering incidental findings. We further analyzed the within-site consistency in handling incidental findings for the six sites where we had collected four or more consent forms. We identified variability in five out of six sites we examined (Table 5); only one site (site 2) used a single strategy consistently (strategy C).

**Table 4:** Strategies proposed to handle incidental findings (IF) in approved consent forms

	Strategies identified (A to M)												
**Attributes of strategies**	**A**	**B**	**C**	**D**	**E**	**F**	**G**	**H**	**I**	**J**	**K**	**L**	**M**

Subject has the choice to be informed of IF	N	Y	N	?	?	?	N	N	N	N	Y	N	N
Scans will be reviewed by a medical specialist	N	?	N	N	N	Y	?	?	Y	Y	Y	N	N
Subject will be directly informed of findings	Y	Y	Y	Y	?	?	Y	Y	N	Y	N	N	N
Subject's physician will be informed of findings	Y	Y	Y	Y	Y	Y	?	N	Y	Y	Y	Y	N
Subject has the choice for the physician to be informed	Y	Y	Y	Y	Y	Y	?	N	N	N	Y	N	N
Physician must be informed (subject has no choice)	N	N	N	N	N	N	?	N	Y	Y	N	Y	N
No mention of option to inform or not physician	N	N	N	N	N	N	?	Y	N	N	N	N	N
Subject will be invited for follow-up scan in case of IF	N	N	Y	Y	N	N	?	N	N	N	N	N	N

*Total frequency of different strategies to handle IF*	6	2	4	2	1	1	2	1	4	1	4	1	14

Y = yes (attribute is present)

N = no (attribute is not present)

? = uncertainty or ambiguity in presence of the attribute

**Table 5:** Within site variability in handling incidental findings in approved consent forms

Site	Number of consent forms per site	Incidental finding strategies employed (see Table 4) per site
Site 1	8	A, B, M
Site 2	5	C
Site 3	4	D,E,F
Site 4	10	G, H, M
Site 7	5	I, K, M
Site 9	7	I, K, L, M

## Discussion

The overall efficacy and performance of ethics review systems have not been investigated thoroughly in spite of their important role of protecting research subjects and ensuring the continuity of the research enterprise. Canada has a long history of research ethics governance and has inspired research ethics review in other countries thus evidence on its workings may have national and broader international relevance.[14] The results of our analysis of approved consent forms for MRI and fMRI research in the Canadian context provides some perspectives on the ethics review process, a process that has been reported as challenging for neuroscientists to understand and navigate.[15,16] There appears to be significant variability and inconsistency in important areas in neuroimaging research such as risk reporting and the management of incidental findings. For example, we found evidence of substantial between-site and within-site variability in the strategies approved to handle incidental findings. There were also significant variations in the disclosure of risks associated with MRI and fMRI consent forms, especially with respect to psychological risks and risks associated with dizziness, discomfort, stress, and fatigue. We also encountered a few forms where the risks of MRI were downplayed (e.g., "no-risk" statements) and incidental findings were described as a benefit.

There are important limitations to keep in mind in this retrospective study of approved consent forms. First, the study is based on a convenience sample and we relied on researchers to send us their most recent approved consent forms. This could have induced some biases if researchers did not conform to this instruction (e.g., if they sent us their "best" or "worse" consent forms). Getting access directly to REB-approved files would have been another route but this has been reported to be difficult in other studies on MRI research.[17] Second, we did not manage to collect forms from all sites in representative proportions and therefore our results should not be interpreted as an exact representation of the Canadian situation. Third, our focus in this paper is to report observations on the research ethics process based on the examination of informed consent forms. This could be criticized since research ethics, and even informed consent, involves much more than the informed consent form itself. The presentation of the consent form is only a portion of the consent process and the verbal exchange between the participant and the researcher introduces several other variables that are unaccounted for in this specific study. However, a focus on this aspect of ethics review allows us to gain insights on a deliverable of ethics review that has perhaps been vested with the most practical and academic energies. Finally, another limitation consists of our focus on a typically minimal-risk research area and the potential generalizability of our findings. On the one hand, REBs may not consider as extensively minimal risk research as they do with invasive and riskier research. This would be consistent with the proportionate approach of the TCPS which advocates adjusting the level of ethics review scrutiny to the level of risk of a study. On the other hand, MRI and fMRI make compelling cases to examine if a relatively safe and minimal risk area is dealt with adequately or not.

Although it's easy to conclude that REBs could be faulty for any variability and inconsistency observed in the process or final approved ethics documents, it is worth reiterating that research ethics and ethics review are shared responsibilities and that ethics governance systems are comprised of distinct key stakeholders such as researchers, REBs, funding bodies, and academic institutions.

One possible partial explanation for the variability and apparent inconsistency we observed could be explained by sensitivity of REBs to local concerns. REBs were created to respond to local concerns of patient and subject communities as well as local administrations and cultures. Accordingly, reported variability could reflect the fact that the institutional and research context varies between sites (e.g., having access to clinicians or not in strategies deployed to handle incidental findings). However, variability is somewhat hard to justify on this basis, given that different strategies for incidental findings were approved by the same institution. Further, some variability in the reporting of risks seems hard to understand on the basis of local concern, considering subject protection and information are key goals of all REBs and research ethics policy and that the basic risks of MRI and fMRI do not vary in essence between sites.

Another partial explanation relates to lack of cohesion and consensus, across Canadian researchers and REBs, about the risks involved in MRI and fMRI studies.[17,18] The causes of this could be manifold: lack of a common and standard understanding of the risks related to MRI by researchers and REBs, lack of access to knowledge of the risks of MRI (e.g., review papers on risks of MRI, MRI guidelines of the Food and Drug Administration or Health Canada); inconsistent application of commonly accepted knowledge of the risks of MRI; lack of REB resources to review the specificities of the risks of novel technologies; lack of consensus between researchers and REBs on the risks of MRI that should be reported to subjects. A previous study using a mock review of fMRI results suggested that although REBs tend to use similar criteria, they can arrive at radically different decisions.[19]

From a research ethics standpoint, the variability and inconsistencies we observed are important because the full disclosure of risks remains both legally mandatory and ethically required in Canada. At least two Canadian court decisions *Halushka *v. *University of Saskatchewan *in 1965 and *Weiss *v. *Solomon *in Quebec have highlighted these obligations.[7,20,21] The variability we encountered regarding some basic risks of MRI and fMRI suggest that not all subjects receive similar information to consent to research participation (e.g., variability in reporting physical and psychological risks). This means that subjects may not be fully informed based on Canadian standards and that protocols and informed consent forms are being accepted with some lacunae. The amount of variation found in risk disclosure of a common research tool points to potential problems with the ability of current research ethics governance systems to deal with risks in novel neuroscience techniques like fMRI. Additionally, there is now mounting evidence of challenges plaguing ethics governance based on local ethics review processes like those in place in Canada, the USA, the UK, and Australia.[22-26] For example, some studies and analyses have suggested that practical shortcomings and variability in the process undermine the trust that researchers place in REBs and creates difficulties for collaboration and open communication.[5,16] REBs are also struggling to deal with demands of the research community for both comprehensive in-depth and expedient review.[27]

Administrative hurdles coupled with an increasingly large burden of responsibility placed on researchers are not reasons to take ethics short-cuts or to dismantle research ethics as we know them. But the combination of sub-optimal performance in dealing with ethics issues clearly and consistently along with a significant practical burden should make us reassess if our energies are well served and if other ways of dealing with the requirement of a proper assessment of ethical issues should be considered. Based on several reports about the challenges encountered in current research ethics governance systems, the status quo option appears hard to defend in research-intensive countries with similar ethics systems like the US, Canada, and the UK. Several reports on ethics governance in these countries indicate structural problems that will not be resolved by only more of the same practice and policy.[28] Different strategies could be adopted to revise current research ethics governance. In Canada, a revision process of the TCPS is taking place.[29] However, little in the proposed revisions seems to address the practical or administrative issues that may make the ethics review process difficult or inconsistent. The focus is on reshaping the policy itself, not its implementation by REBs and research institutions.[30] Additionally, we lack an evidence-based approach to making revisions that would be commanded by both evidence about the existence of problems and evidence about the efficiency of proposed revisions.[5]

## Conclusion

The time to engage in open-minded discussions about changes to align the priorities of ethics governance to subject protection and information with administrative responsiveness and accountability seems ripe. We need additional research in this domain to provide solid evidence on the lacunae of ethics review and the efficiency of proposed solutions before implementation. Acknowledging the shortcomings of our best efforts within the current systems is a first step in preparing new directions and reflections crafted by the different stakeholders of research ethics.

## Competing interests

The authors declare that they have no competing interests.

## Authors' contributions

ER conceived the study. All authors participated in its design. CD collected the raw data. NP and WA coded the data. NP helped in the coordination of the study. NP and ER performed data analysis. NP and ER drafted the manuscript. All authors were involved in the editing of the paper. All authors have read and approved the final manuscript.

## Pre-publication history

The pre-publication history for this paper can be accessed here:

http://www.biomedcentral.com/1472-6939/12/1/prepub
